# TT virus hepatitis with extrahepatic and hepatic manifestations - case report

**DOI:** 10.1186/1471-2334-13-S1-P71

**Published:** 2013-12-16

**Authors:** Victor Pântea, Gheorghe Plăcintă, Valentin Cebotarescu, Lilia Cojuhari, Valentina Smeşnoi

**Affiliations:** 1 Department of Infectious Diseases, Faculty for Continuing Medical Education, Nicolae Testemițanu State Medical and Pharmacy University, Chişinău, Republic of Moldova; 2 Toma Ciorbă Clinical Hospital for Infectious Diseases, Chişinău, Republic of Moldova; 3 Chiril Draganiuc Institute of Phthisiopneumology, Chişinău, Republic of Moldova

## Background

TT virus (TTV) hepatitis is an infectious disease which can have both hepatic and extrahepatic manifestations, and it is transmitted prevalently parenterally, and rarely by enteral way. The prevalence of TTV in donors in the USA is 1%, from 1.9-10% in Great Britain, in Japan - 12%, in Thailand 7-30%, in Russia 10-20%, and in Brazil 62%. In patients with cryptogenic cirrhosis, the TTV was revealed in 15% of the cases, and 27% in fulminant hepatitis, 40% in hemophiliacs, 47.9% in those with hemodialysis.

## Case report

We present a clinical case with viral TTV hepatitis revealed in a young person born in 1987, from a village in the northern part of Moldova. He fell ill at the beginning of January 2012 being absolutely healthy when a rash appeared, an erythematic eruption on the cheeks and the nasal pyramid, moderate pains in the joints and abdominal discomfort. At the moment of falling ill he worked in Russia at the construction of the Olympic Complex in Soci. He consulted a doctor which suspected him of systemic lupus erythematosus, but it was not confirmed in the laboratory, and no treatment was administered. The symptomatology of the disease disappeared. But in May (5 months from the time the first symptoms appeared) the pains in the joints reappeared, mostly in the big joints. He addressed the Infectious Diseases Clinic of the State Medical and Pharmaceutical University “Nicolae Testemițanu” and during the objective examination, a moderate hepatomegaly was confirmed at an ultrasound examination: the right lobe -14.6 cm, the left lobe – 4.6 cm, the spleen- 11.8x5.6 cm. He underwent a serological, biochemical and an instrumental (FibroScan) investigation. We also investigated the viral hepatitis markers: HBsAg, HBeAg, anti-HBc IgM, total anti-HBc, anti-HBe, anti-HBs, anti-HCV IgM, total anti-HCV, anti-HDV IgM, total anti-HDV, anti-HEV IgM, anti-HEV IgG, anti-VCA IgM, anti-VCA IgG, anti-CMV IgM, anti-CMV IgG and anti-E2HGV – negative. Biochemical investigations: ALAT - 83.9 U/L, ASAT - 76 U/L, total bilirubin - 18.6 µmol/L, direct - 2.6, indirect - 16.0. Instrumental investigations (FibroScan): the average coefficient of hepatic elasticity was 8.8 kPa, which corresponds to F1-F2 stage of hepatic fibrosis (according to the Metavir scale). The diagnosis of the viral hepatitis with TT virus has been confirmed by a biological molecular test DNA-TTV, a qualitative-positive test (the Litex laboratory in Moscow, 16.05.2012).

